# Glycogen Synthase Kinase-3β Inhibitor VP3.15 Ameliorates Neurogenesis, Neuronal Loss and Cognitive Impairment in a Model of Germinal Matrix-intraventricular Hemorrhage of the Preterm Newborn

**DOI:** 10.1007/s12975-023-01229-2

**Published:** 2024-01-17

**Authors:** Isabel Atienza-Navarro, Angel del Marco, Pilar Alves-Martinez, Maria de los Angeles Garcia-Perez, Alvaro Raya-Marin, Isabel Benavente-Fernandez, Carmen Gil, Ana Martinez, Simon Lubian-Lopez, Monica Garcia-Alloza

**Affiliations:** 1 Division of Physiology, School of Medicine, University of Cadiz, C/Dr. Marañon 3, 3rd Floor, 11002 Cadiz, Spain; 2 Biomedical Research and Innovation Institute of Cadiz (INiBICA) Research Unit, Puerta del Mar University Hospital, Cadiz, Spain; 3 Area of Pediatrics, Department of Child and Mother Health and Radiology, School of Medicine, University of Cadiz, Cadiz, Spain; 4 Section of Neonatology, Division of Pediatrics, Puerta del Mar University Hospital, Avda. Ana de Viya sn, 11007 Cadiz, Spain; 5 Centro de Investigaciones, Biologicas Margarita Salas-CSIC, Ramiro de Maeztu 9, 28040 Madrid, Spain; 6 Centro de Investigaciones Biomedicas en Red en Enfermedades Neurodegenerativas (CIBERNED), Instituto de Salud Carlos III, Avda. Monforte de Lemos 3-5, 28029 Madrid, Spain

**Keywords:** Preterm newborn, Germinal matrix-intraventricular hemorrhage, VP3.15, Neurodegeneration, Neurogenesis, Cognition

## Abstract

Advances in neonatology have significantly reduced mortality rates due to prematurity. However, complications of prematurity have barely changed in recent decades. Germinal matrix-intraventricular hemorrhage (GM-IVH) is one of the most severe complications of prematurity, and these children are prone to suffer short- and long-term sequelae, including cerebral palsy, cognitive and motor impairments, or neuropsychiatric disorders. Nevertheless, GM-IVH has no successful treatment. VP3.15 is a small, heterocyclic molecule of the 5-imino-1,2,4-thiadiazole family with a dual action as a phosphodiesterase 7 and glycogen synthase kinase-3β (GSK-3β) inhibitor. VP3.15 reduces neuroinflammation and neuronal loss in other neurodegenerative disorders and might ameliorate complications associated with GM-IVH. We administered VP3.15 to a mouse model of GM-IVH. VP3.15 reduces the presence of hemorrhages and microglia in the short (P14) and long (P110) term. It ameliorates brain atrophy and ventricle enlargement while limiting tau hyperphosphorylation and neuronal and myelin basic protein loss. VP3.15 also improves proliferation and neurogenesis as well as cognition after the insult. Interestingly, plasma gelsolin levels, a feasible biomarker of brain damage, improved after VP3.15 treatment. Altogether, our data support the beneficial effects of VP3.15 in GM-IVH by ameliorating brain neuroinflammatory, vascular and white matter damage, ultimately improving cognitive impairment associated with GM-IVH.

## Introduction

Approximately 15 million babies are born premature (before 37 gestational weeks) every year worldwide, accounting for ≈11% of all births [1]. Whereas mortality rates associated with prematurity have been reduced in recent decades due to advances in neonatology, complications of prematurity are still a societal and medical burden. Among these, germinal matrix-intraventricular hemorrhage (GM-IVH) is one of the most relevant complications of prematurity and the most common intracranial hemorrhage in preterm infants (PT).

The germinal matrix is a richly vascularized region around the ventricles and a source of neural progenitors that starts to involute from gestational week 28 until it disappears at 36 gestational weeks [2]. Therefore, when PT are born, the germinal matrix is an immature structure that is prone to bleeding due to the limited development of the central nervous system (CNS) as well as the hemodynamic instability of PT and their difficulty to regulate cerebral blood flow and fluctuations, among others (for review, see [3]).

GM-IVH is usually classified into 4 degrees depending on the severity. Grades I and II are considered mild GM-IVH (in grade I the hemorrhage is limited to the subependymal germinal matrix and in grade II there is ventricular hemorrhage without ventricular dilation) and grades III and IV are severe GM-IVH (grade III there is ventricular hemorrhage with ventricular dilation and in grade IV there is parenchymal hemorrhage associated with periventricular infarctions) [4]. However, grade IV GM-IVH is no longer considered just a propagation of the original hemorrhage but a result of the obstruction of the venous drainage that leads to venous infarction and hemorrhage of the surrounding tissue as periventricular hemorrhagic infarction [5, 6].

Almost half of low-birth-weight (under 2500 g) PT will develop GM-IVH (for review [7]). Importantly, many of these babies will present structural and functional brain alterations that include ventricle enlargement, white matter and vascular damage or neuroinflammatory alterations. As a result, the risk of suffering cognitive or motor complications, developmental delay, cerebral palsy or psychiatric disorders is significantly increased in PT with GM-IVH [3]. In this sense, PT with severe GM-IVH will commonly suffer developmental delay [8, 9] and some studies show that up to 42% of very PT with periventricular hemorrhage will develop cerebral palsy [10]. Moreover, while the long-term neurodevelopmental complications seem out of the question in severe GM-IVH, it has also been shown that in very low body weight infants even mild GM-IVH will increase by twofold the need of special education [8].

At present, GM-IVH has no successful treatment. Different therapeutic options have been explored, including prenatal corticoid administration [11]. Other studies have included postnatal treatments with indomethacin [12, 13], caffeine [14], vitamin E [15], AMPA-kainate inhibitors [15, 16] or erythropoietin [17], among others. Nevertheless, the success of these approaches is limited and GM-IVH has no definitive treatment. Therefore, GM-IVH patients are in great need of new alternatives that may reduce brain damage and behavioral complications. In this sense, glycogen synthase kinase-3β (GSK-3β) inhibitors have been assessed in different neurodegenerative diseases due to their implication in proinflammatory-anti-inflammatory balance as well as in neurodegenerative pathways, tau phosphorylation or neurogenesis [18–20]. Among GSK-3β inhibitors, previous studies have shown the neuroprotective effects of VP3.15. This is a small heterocyclic molecule of the 5-imino-1,2,4-thiadiazole family with dual effects, as a GSK-3β inhibitor but also as a phosphodiesterase 7 (PDE7) inhibitor, alleviating neuroinflammation and neuronal loss. VP3.15 reduces inflammation in experimental autoimmune encephalomyelitis [21]. It also limits neurodegeneration in a model of retinitis pigmentosa [22] and promotes remyelination in models of multiple sclerosis [23, 24].

Given the described neuroprotective capacity of VP3.15 and the neuronal loss, neuroregeneration compromise and inflammation associated with GM-IVH, we administered VP3.15 to an animal model of GM-IVH induced by intraventricular unilateral administration of collagenase to P7 CD1 mice [14, 17, 25]. We have previously characterized this model [25] and, whereas interindividual differences are observed, this approach generally reproduces the most severe version of the disease and ventricular hemorrhage with ventricular dilation, as well as periventricular hemorrhagic infarction are commonly observed.

We studied the effects of VP3.15 in the short (P14) and long term (P110) by analyzing neuropathological alterations commonly observed in patients with GM-IVH, including the presence of hemorrhages and microglia recruitment, as well as neuronal damage and neurogenesis alterations. The effects of VP3.15 on white matter have also been addressed as well as the overall effects on cognition. Importantly, we also analyzed plasma gelsolin (p-GLS), a calcium-dependent actin regulatory protein that might be a biomarker for subarachnoid hemorrhage-related complications in adults [26] and neonates with hypoxia-ischemic encephalopathy [27]. Altogether, our data support a role for VP3.15 in reducing brain neuroinflammatory damage after GM-IVH by ameliorating and improving associated cognitive impairment, ultimately contributing to a better understanding of the pathology of GM-IVH and the beneficial effects of VP3.15.

## Material and Methods

### Animals and Treatments

**Fig. 1:**
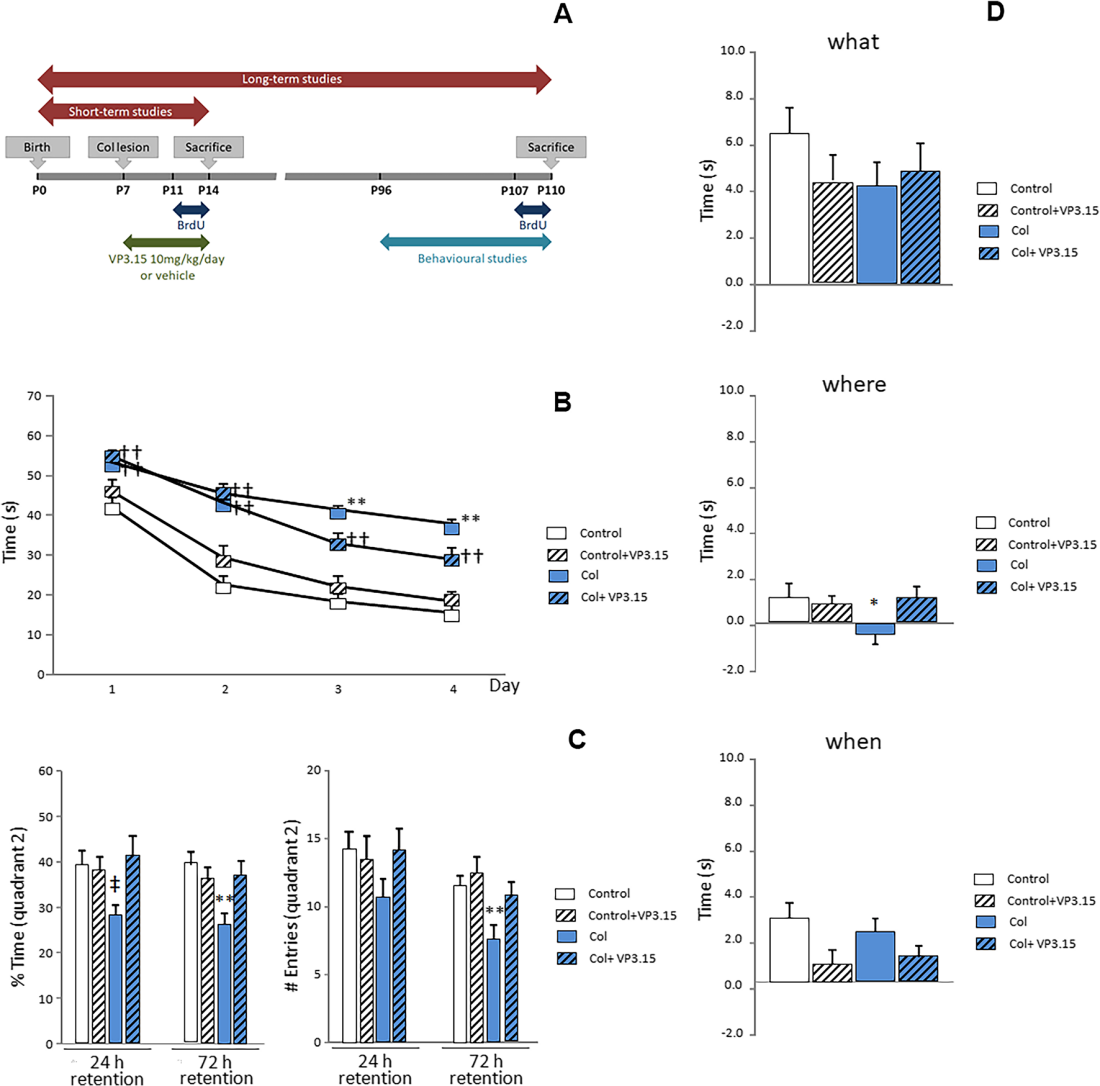
VP3.15 ameliorates cognitive impairment in animals with GM-IVH. **a** Timeline showing relevant timepoints of the experimental set up. Collagenase (Col), 5-bromo-2-deoxyuridin (BrdU). **b** Learning was affected in animals after Col lesions, and VP3.15 treatment significantly reduced the time required to locate the platform during the acquisition phase in the MWM (day 1 [F_(3,337)_ = 10.57, ††*p* < 0.01 vs. Control and Control-VP3.15], day 2 [F_(3,342)_ = 23.92, ††*p* < 0.01 vs. Control and Control-VP3.15], day 3 [F_(3,337)_ = 21.70, **p < 0.01 vs. rest of the groups, ††*p* < 0.01 vs. Control and Control-VP3.15], day 4 [F_(3,338)_ = 20.43, **p < 0.01 vs. rest of the groups, ††*p* < 0.01 vs. Control and Control-VP3.15]). **c** VP3.15 also increased the time the animals spent in quadrant 2 (where the platform used to be located during the acquisition phase) both 24 h [F_(3,77)_ = 2.78, ‡*p* = 0.037 vs. Control] and 72 h [F_(3,78)_ = 5.03, **p = 0.003 vs. rest of the groups] after completing the acquisition phase. Similarly, the number of entrances in quadrant 2 was higher in animals with GM-IVH that received VP3.15 (24 h [F_(3,77)_ = 1.74, *p* = 0.165], 72 h [F_(3,80)_ = 5.29, **p = 0.002 vs. rest of the groups]). Data are representative of 16–25 mice (Control *n* = 25, Control + VP3.15 *n* = 16, Col *n* = 24, Col + VP3.15 *n* = 22). **d** Episodic memory was affected after Col injection. Differences reached statistical significance for the “where” paradigm, and VP3.15 treatment significantly ameliorated this situation (“what” [F_(3,230)_ = 0.945, *p* = 0.419]; “where” [F_(3,236)_ = 3.80, *p = 0.011 vs. rest of the groups]; “when” [F_(3,242)_ = 0.40, *p* = 0.068]). Data are representative of 17–23 mice (Control *n* = 25, Control + VP3.15 *n* = 17, Col *n* = 23, Col + VP3.15 *n* = 21)

GM-IVH was induced to seven-day-old (P7) CD1 mice by intraventricular unilateral injection of collagenase (Col) (purified collagenase VII, batch C0773-1.5KU, Sigma Aldrich, St Louis, MO, USA) in 1 µl of TESCA (TES buffer 50 mM, calcium chloride anhydrous 0.36 mM) as described [25]. Since Col is obtained from the culture filtrate of *Clostridium histolyticum*, the enzymatic activity might depend on the actual batch used for the study. Briefly, animals were anesthetized with isoflurane, and Col was administered in the right hemisphere by stereotaxic injection (David-Kopf, CA, USA) at 0.1 μl/min for 10 min using a 10 μl Hamilton syringe (Hamilton Company, USA). The coordinates from bregma were -1 mm mediolateral and -3 mm anteroposterior. The dorsoventral coordinate was + 4 from the meninges. To avoid aspiration, the needle was left in the lesion site for 5 min once the infusion was completed. Sham animals underwent the same procedure but received 1 µl of TESCA. A naive group of animals did not undergo any surgical procedures. After recovery, the mice were returned to their home cages with their mothers. Animals were treated with VP3.15, provided with CIB-CSIC and synthesized according to previously described procedures [28], 10 mg/kg/day in DMSO (10%) in PBS for 7 consecutive days (P7-P13), and non-treated animals received the vehicle. First administration of VP3.15 was performed 10 min after concluding the surgery and every 24 h afterwards. Short-term studies took place at P14, and long-term studies were performed in young adults at P110. Two weeks before sacrifice at P110, animals underwent behavioral assessment to analyze cognition and motor activity. Three days prior to sacrifice, at P14 or P110, the animals received a daily dose of 5-bromo-2-deoxyuridin (BrdU) (70 mg/kg). Animals were randomly assigned to each experimental group, and analysis was performed blindly by the researcher. Timeline of experimental procedures is presented in Fig. 1a. All experimental procedures were approved by the Animal Care and Use Committee of the University of Cadiz and Junta de Andalucia in accordance with the guidelines for the care and use of experimental animals (European Commission Directive 2010/63/UE and Spanish Royal Decree 53/2013).

### Morris Water Maze (MWM)

Behavioral assessment in the MWM commenced at P96. Animals were tested in a round pool of 90 cm (water temperature 21 ± 2 °C) with visual clues located in the external part of each virtual quadrant. Acquisition took place for 4 consecutive days (4 trials/day), and mice were allowed to swim for 60 s to locate the hidden platform in quadrant 2. When the animal reached the platform, it was allowed to stay for 10 s. If the animal did not find the platform, it was manually located for 10 s. The retention test took place 24 and 72 h after completing the acquisition phase and consisted of a single trial without the platform. Time to locate the platform during the acquisition phase and the number of entries as well as time spent in quadrant 2 and speed in the retention phase were analyzed using Smart software (Panlab, Spain).

### New Object Discrimination Test (NOD)

Motor activity was analyzed 24 h after completing the MWM test. Animals were placed in a rectangular box (length 44 cm × width 22 cm × height 40 cm). For habituation purposes, the next day, animals were exposed for 5 min to two objects (red cylinder and yellow trapezoid) that were not used again during the experiment. On day 3, mice performed two sample trials and a test trial. In the first trial, mice explored 4 copies of a novel object (navy balls) for 5 min in a triangular configuration. After 30 min, the mice received a 5 min sample trial with 4 new objects (red cones) in a quadrangular shape. Thirty minutes later, the animals received a test trial with two copies of the object from sample trial 2 (“recent” objects) placed in the same position and two copies of the object from sample trial 1 (“familiar” objects), with one placed in the same position (“familiar non-displaced” object) and the other in a new position (“familiar displaced” object). Episodic memory for “what”, “where” and “when” paradigms was analyzed as described [29]. "What" was the difference in time exploring familiar and recent objects, "where" was the difference in time exploring displaced and non-displaced objects and "when" was the difference between time exploring familiar non-displaced and recent non-displaced objects.

### Tissue and Sample Processing

Animals were sacrificed by intraperitoneal pentobarbital overdose (120 mg/kg). Blood samples (P14 and P110) were collected in heparinized tubes, and after centrifuging for 10 min (6500 rpm), plasma was frozen at − 80 °C until use. Brains were harvested and weighed. The ipsilateral cortex, hippocampus and striatum were dissected in half of the animals and snap frozen for ELISA studies. The brains from the remaining animals were fixed in 4% paraformaldehyde for 2 weeks. Brain tissue from P14 mice was snap frozen using isopentane (277,258-100ML, Merk) cooled with liquid nitrogen before the tissue was sectioned. A cryostat was used to obtain 30 μm-thick coronal sections for staining and immunostaining procedures.

### Cresyl Violet Staining

Brain morphology was analyzed, and ipsilateral hemisection, cortex, hippocampus and ventricle sizes were measured. Six coronal Sects. (1.5, 0.5, 0.0, -1.5, -2.0 y -2.5 mm from bregma) were stained for 10 min with cresyl violet (Sigma, St. Louis, MO, USA) solution (0.5% w/v) as described [17]. Sections were analyzed in an optical Olympus Bx60 microscope (Olympus, Tokyo, Japan) with an Olympus DP71 camera (Olympus, Tokyo, Japan). Cell F software (Olympus, Hamburg, Germany) was used to acquire the images, and ipsilateral hemisection, cortex, hippocampus and lateral ventricle sizes were measured using Adobe Photoshop Elements and ImageJ software.

### Prussian Blue Staining

Hemorrhage size, density and burden were assessed in the cortex, hippocampus, SVZ and striatum by Prussian blue staining and neutral red counterstaining as described [30]. Six sections contiguous to those used for cresyl violet staining were imaged with an Olympus Bx60 microscope (Olympus, Tokyo, Japan) with an Olympus DP71 camera (Olympus, Tokyo, Japan). Hemorrhage burden (% of area covered by hemorrhages) was analyzed in the cortex, hippocampus, SVZ and striatum using Adobe Photoshop Elements and ImageJ software.

### Immunohistochemical Studies: NeuN, Iba1 + , BrdU and Doublecortin (DCX)

Neurons were labeled with anti-NeuN (Abcam, Netherlands, ab279297) (1:500), and microglia were labeled with anti-Iba1 (Wako, Osaka, Japan, 019–19741) (1:1000) overnight at 4 °C as described [31]. Alexa Fluor goat anti-rabbit 594 (Abcam, Netherlands, ab150160) and Alexa Fluor goat anti-rabbit 488 (Invitrogen, Carlsbad, CA, USA, A11008) (1:1000) were used as secondary antibodies, followed by counterstaining with DAPI 1 mg/ml (Sigma, St. Louis, MO, USA, D9542) (1:3000) for 10 min. We analyzed the percentage of NeuN-positive cells (normalized by total cells stained with DAPI) in the cortex, hippocampus and SVZ [32] using ImageJ software [33]. Microglial burden (% of area covered by Iba1^+^ cells) was also analyzed in the cortex, hippocampus and SVZ.

BrdU and DCX immunostaining was analyzed in the SVZ, the subgranular zone of the DG and the cortex as described [34]. Briefly, sections were pretreated with SSC buffer (S6639, Fisher Scientific, UK) and formamide (BP227, Sigma, St. Louis, MO, USA) (1:1) (2 h at 65 °C). Then, the sections were washed, incubated in 2 N hydrochloric acid (131,020, Panreac AppliChem) (30 min at 37 °C), and washed in 25 mM borate buffer (B-9876, Sigma, St. Louis, MO, USA) (pH = 8.4). The sections were blocked with 0.1% Triton-X (BP151-100, Fisher Scientific, UK) in 2.5% BSA (A7906, Sigma, St. Louis, MO, USA) (1 h at RT). Sections were incubated overnight at 4 °C in primary antibodies anti-BrdU 1:100 (Abcam, Netherlands, ab6326) and anti-DCX 1:200 (Abcam, Netherlands, ab18723). Alexa Fluor goat anti-rat 488 (Abcam, Netherlands, ab150157) and Alexa Fluor goat anti-rabbit 594 (Abcam, Netherlands, ab150080) conjugated antibodies (1:1000) were used as secondary antibodies. Images of 30 µm in depth with a Z-step size of 2 μm were obtained using a × 20 objective on a Zeiss LSM 900 Airyscam 2 confocal microscope (Zeiss, Oberkochen, Germany). Images up to 200 µm from the ventricle lumen were acquired to fully cover the SVZ and merged using Photoshop Elements software. The number of individual BrdU^+^ cells was quantified in the SVZ as previously described [34, 35]. Since the number of DCX^+^ cells is very high in the SVZ^+^, delimiting individual cell cytoplasms could not be done in a reliable manner. Therefore DCX burden (percentage of area covered by DCX-positive cells) was quantified in the SVZ, using Image J free software. The number of individual BrdU^+^ cells in the SVZ was quantified using Image J. The BrdU/DCX ratio was also measured in the SVZ. The number of individual DCX^+^ and BrdU^+^ cells, as well as BrdU^+^/DCX^+^ were also quantified in the subgranular zone of the DG using ImageJ software. DCX^+^, and BrdU^+^ cells in the cortex were quantified *de visu* using an Olympus Bx60 fluorescence microscope (Olympus, Tokyo, Japan) coupled to an Olympus DP71 camera and MMIcellTools software.

### Phospho-tau and Total Tau Levels

Colorimetric ELISA kits were used to measure total tau (KMB7011, Invitrogen, Thermo-Fisher Scientific, USA) and phospho-tau [pS199] (KMB7012, Invitrogen, Thermo-Fisher Scientific, USA) levels in the cortex and striatum according to the manufacturer's instructions [14]. Briefly, between 5 and 10 mg of tissue were homogenized in 50 µl of homogenization buffer (5 M guanidine-HCl diluted in 50 mM Tris) with protease and phosphatase inhibitor cocktail for 3–4 h at RT, shaking every 20–30 min until complete homogenization. After centrifugation at 14,500 × g for 20 min at 4 °C, supernatants were collected, and absorbances were measured at 450 nm in a spectrophotometer (MQX200R2, Biotek instruments, Burlington VT, USA). Phospho-tau/total-tau ratios in pg/mg tissue were calculated, and the results were expressed as a percentage of control values.

### Neurofilament Light (Nfl), MBP and p-GLS

The content of Nfl was determined by ELISA (NF-light ELISA, Ref. 10–7001, UmanDiagnostics AB) in the cortex following the manufacturer´s indications. Five to 10 mg of tissue were homogenized in 75 µl of PBS on ice and centrifuged at 3000 rpm for 20 min at 4 °C. Thereafter, the supernatants were collected and diluted in kit buffer. The absorbances were measured at 450 nm in a spectrophotometer (MQX200R2, Biotek instruments, Burlington VT, USA). Nfl levels in the cortex were calculated, and the results were expressed as pg/mg tissue.

Similarly, MBP was also quantified by ELISA (Biorbyt, ORB40937896). Five to 10 mg of tissue were selected and homogenized in 70 µl of PBS. Samples were centrifuged at 4 °C (5 min at 5000 g), and supernatants were collected and diluted 1:2. After appropriate incubations, the plate was read on a spectrophotometer (MQX200R2, Biotek instruments, Burlington VT, USA) at 450 nm. The results were expressed in pmol/g tissue, and provide information on myelination in the cortex.

Plasma samples were diluted 1:500, and p-GLS levels were quantified by colorimetric ELISA kits (ABIN367662, Antibodies-online) following the manufacturer's recommendations. Data were expressed as percentaje of control values.

### Statistical Analysis

Two-way ANOVA (groupXday) was performed to analyze the acquisition phase in the MWM test. One-way ANOVA for independent samples, followed by Tukey’s b or Tamhane tests as required, was performed in all experimental studies. SPSS v.24 software was used for all statistical analyses. Data are expressed as the mean ± SEM. Differences were considered statistically significant for *p* values < 0.05 and “vs. rest of the groups” was used to identify differences between a group and all the other groups included in the specific determination.

## Results

### Cognitive Impairment is Ameliorated by VP3.15 in Animals with GM-IVH

Learning and memory were impaired in animals with GM-IVH, and we observed a significant timeXgroup effect when we analyzed the acquisition phase in the MWM [F_(9,1354)_ = 1.99 *p* = 0.037]. Individual daily assessment revealed a slight improvement in animals with GM-IVH after VP3.15 treatment, as training advanced (Fig. 1b).

Similarly, animals after Col lesions were significantly affected when the time spent in quadrant 2 or the number of entrances in quadrant 2 was compared 24 and 72 h after the completion of the acquisition phase. Nevertheless, VP3.15 treatment ameliorated this situation, and GM-IVH animals on VP3.15 showed similar performances to those observed in control mice (Fig. 1c). No differences among groups were observed for the “what” and “when” paradigms in the NOD test. However, episodic memory for the “where” paradigm was affected in animals with GM-IVH, and VP3.15 significantly improved the performance of treated mice (Fig. 1d).

Swimming velocity was not affected in any of the groups under study at 24 h (Control = 25.53 ± 0.97, Control + VP3.15 = 27.34 ± 0.86, Col = 22.61 ± 1.70, Col + VP3.15 = 24.11 ± 1.29 cm/s; [F_(3,83)_ = 2.21, *p* = 0.101)] or 72 h (Control = 25.20 ± 0.8, Control + VP3.15 = 27.22 ± 0.95, Col = 22.94 ± 1.31, Col + VP3.15 = 23.53 ± 1.48 cm/; [F_(3,83)_ = 2.21, *p* = 0.101]).

### VP3.15 Reduces the Presence of Hemorrhages in Animals with GM-IVH

**Fig. 2:**
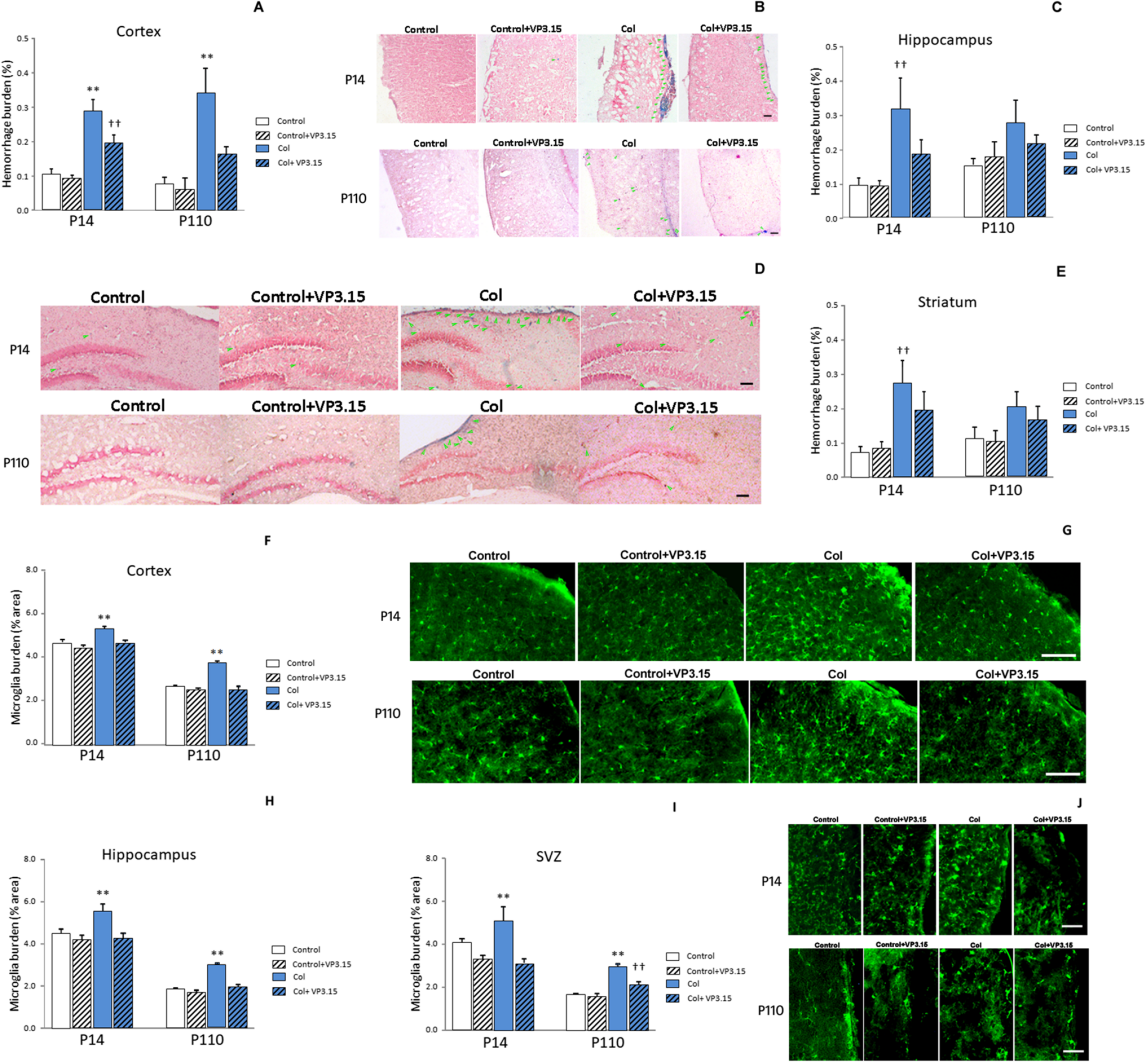
VP3.15 reduces the presence of hemorrhages and Iba1^+^ microglia in animals with GM-IVH. **a** Hemorrhage burden was higher in mice with GM-IVH, and VP3.15 significantly reduced the presence of hemorrhages in the cortex at P14 [F_(3,94)_ = 13.81, **p < 0.01 vs. rest of the groups, ††*p* < 0.01 vs. Control and Control + VP3.15] and completely reversed the effect of the lesions at P110 [F_(3,119)_ = 9.22, **p < 0.01 vs. rest of the groups]. **b** Representative images of cortical regions stained with Prussian blue. Green arrows point at individual hemorrhages. Scale bar = 100 µm. **c** Increased hemorrhage burden in the hippocampus was also reduced by VP3.15 in the short term [F_(3,50)_ = 6.04, ††*p* < 0.01 vs. Control and Control + VP3.15], and a similar profile was observed in the long term, although the differences did not reach statistical significance [F_(3,49)_ = 2.15, *p* = 0.105]. **d** Illustrative images of hippocampal regions stained with Prussian blue. Green arrows point at individual hemorrhages. Scale bar = 100 µm. **e** VP3.15 also reduced the presence of hemorrhages in the striatum from mice with GM-IVH at P14 [F_(3,42)_ = 4.36, ††*p* = 0.009 vs. Control and Control + VP3.15], although no differences were observed at P110 [F_(3,35)_ = 1.087, *p* = 0.367]. Data are representative of 5–7 mice (P14: Control *n* = 6, Control + VP3.15 *n* = 6, Col *n* = 5, Col + VP3.15 *n* = 7. P110: Control *n* = 6, Control + VP3.15 *n* = 5, Col *n* = 6, Col + VP3.15 *n* = 7). **f** Microglial burden was significantly increased in the cortex after Col lesions, and VP3.15 reduced microglial burden, reaching control values at P14 [F_(3,2418)_ = 1.27, **p < 0.01 vs. rest of the groups]. A similar profile was observed at P110 [F_(3,2836)_ = 97.24, **p < 0.01 vs. rest of the groups]. **g** Illustrative examples of cortical microglia immunostaining for Iba1 in all groups under study. Scale bar = 100 µm. **h** Increased microglial burden in the hippocampus from animals with GM-IVH was also restored to control values at P14, after treatment with VP3.15 [F_(3,365)_ = 5.94, **p = 0.001 vs. rest of the groups]. The induced increase in microglia in the hippocampus at P110, was also limited by VP3.15 [F_(3,470)_ = 19.20, **p = 0.001 vs. rest of the groups]. **i** Microglial burden was also improved in the SVZ at P14 after treatment with VP3.15 [F_(3,292)_ = 9.31, **p = 0.001 vs. rest of the groups]. Microglial burden was also increased in the SVZ by P110, and VP3.15 limited this effect [F_(3,484)_ = 23.21, **p < 0.001 vs. rest of the groups, ††*p* = 0.001 vs. and Control + VP3.15]. Data are representative of 4–7 mice (P14: Control *n* = 5, Control + VP3.15 *n* = 7, Col *n* = 4, Col + VP3.15 *n* = 4. P110: Control *n* = 6, Control + VP3.15 *n* = 6, Col *n* = 5, Col + VP3.15 *n* = 6). **j** Illustrative examples of SVZ microglia immunostaining for Iba1 in all groups under study. Scale bar = 50 µm

We observed an overall increase in the presence of hemorrhages that affected not only the areas closer to the lesion (striatum), but also regions located in more distant areas, like the cortex, in line with previous observations in this model [14, 25]. Similarly, other studies have reported vascular alteration in cortical areas both in patients [36] and in other animal models [37]. Hemorrhage burden (% of area affected by hemorrhages) was significantly increased in the cortex from mice after the lesions. VP3.15 effectively reduced the hemorrhage at P14 and completely reversed this vascular damage at P110 (Fig. 2a and b). We also analyzed the hippocampus, and we observed an increase in hemorrhage burden in animals with GM-IVH in the earliest timepoint that was reduced by VP3.15 treatment. Whereas a similar profile was observed in the long term, the differences were not statistically significant (Fig. 2c and d). Additionally, a similar trend was observed in the striatum, and the increased hemorrhage burden at P14 was ameliorated by VP3.15 (Fig. 2e).

### VP3.15 Reduces Microglial Burden in Animals with GM-IVH

We analyzed the inflammatory process by quantifying the presence of Iba1^+^ cells. We observed a significant increase in Iba1^+^ burden in the cortex from animals with GM-IVH, and VP3.15 alleviated the inflammatory process in the short term. A similar profile was observed when we analyzed the effects of VP3.15 at a late timepoint (Fig. 2f and g). We also observed that the microglial burden was increased in the hippocampus and that VP3.15 reduced the presence of Iba1^+^ cells at P14. Differences were no longer observed at P110 when the treatment was assessed (Fig. 2h). A similar profile was observed in the SVZ, where VP3.15 did not affect microglia burden but successfully reduced the presence of Iba1^+^ cells at P14 (Fig. 2i and j), supporting the anti-inflammatory role of VP3.15.

### Brain Atrophy is Reduced in Animals with GM-IVH treated with VP3.15

**Fig. 3:**
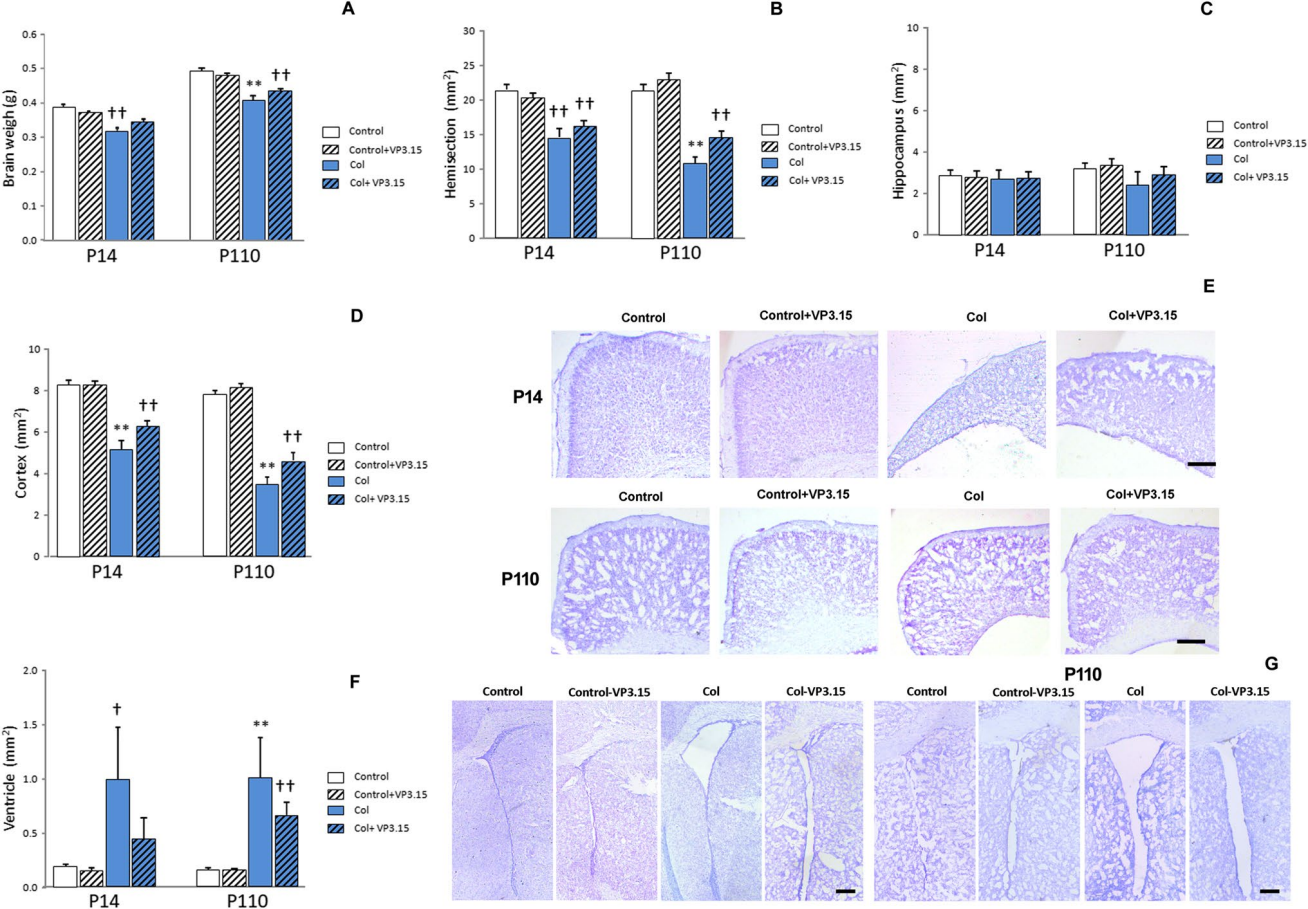
VP3.15 reduces brain atrophy in animals with GM-IVH. **a** Brain weight was significantly reduced in animals with GM-IVH, and VP3.15 limited this effect both at P14 [F_(3,62)_ = 17.66,††*p* < 0.001 vs. Control and Control + VP3.15] and at P110 [F_(3,73)_ = 24.34, **p = 0.027 vs. rest of the groups, ††*p* < 0.001 vs. Control and Control + VP3.15]. The data are representative of 16–24 mice (P14: Control *n* = 17, Control + VP3.15 *n* = 16, Col *n* = 16, Col + VP3.15 *n* = 16. P110: Control *n* = 24, Control + VP3.15 *n* = 16, Col *n* = 23, Col + VP3.15 *n* = 21). **b** GM-IVH reduced hemisection sizes in the early timepoint, and while VP3.15 slightly improved this situation, differences only reached statistical significance in the long term (P14 [F_(3,149)_ = 11.64, ††*p* < 0.01 vs. Control and Control-VP3.15], P110 [F_(3,149)_ = 31.01, **p < 0.01 vs. rest of the groups, ††*p* < 0.01 vs. Control and Control-VP3.15]. **c** No differences among groups were observed when hippocampus size was analyzed at P14 [F_(3,76)_ = 0.178, *p* = 0.918] or P110 [F_(3,36)_ = 0.76, *p* = 0.551]. **d** Nevertheless, cortical size compromise after GM-IVH was significantly ameliorated by VP3.15 both at P14 [F_(3,148)_ = 28.89, **p < 0.01 vs. rest of the groups, ††*p* < 0.01 vs. Control and Control-VP3.15] and P110 [F_(3,147)_ = 57.34, **p < 0.01 vs. rest of the groups, ††*p* < 0.01 vs. Control and Control-VP3.15]. **e** Illustrative example of cortical compromise after GM-IVH and the effect of VP3.15 in limiting cortical thinning in the short and long term. Scale bar = 250 µm. **f** Ventricle enlargement after the lesions was also significantly reduced by VP3.15 treatment both at P14 [F_(3,99)_ = 3.97, †*p* = 0.1 vs. Control and Control-VP3.15] and P110 [F_(3,84)_ = 16.70, **p < 0.01 vs. rest of the groups, ††*p* < 0.01 vs. Control and Control-VP3.15]. The data are representative of 5–8 mice (P14: Control *n* = 8, Control + VP3.15 *n* = 8, Col *n* = 8, Col + VP3.15 *n* = 8. P110: Control *n* = 7, Control + VP3.15 *n* = 5, Col *n* = 7, Col + VP3.15 *n* = 8). **g** Illustrative example of ventricle enlargement and the beneficial effect of VP3.15 at P14 and P110 (+ 0.5 mm anteroposterior from bregma). Scale bar = 250 µm

Brain weight was significantly reduced in animals with GM-IVH at P14, and VP3.15 ameliorated this situation. A similar profile was observed at P110, and whereas VP3.15 did not restore control values, it restored brain weight after Col lesions (Fig. 3a). Microscopic assessment of the brain revealed an overall atrophy that resulted in a reduction of hemisection sizes, analyzed by cresyl violet staining, after GM-IVH. VP3.15 slightly improved this limitation at P14, although differences only reached statistical significance in the later timepoint (Fig. 3b). No significant differences were observed among groups when the hippocampus was analyzed (Fig. 3c), in line with previous observations in this model [14]. Nevertheless, a significant compromise in cortical size was observed after the lesions and VP3.15 limited cortical damage (Fig. 3d and e). Similarly, ventricle enlargement, a marker of brain atrophy commonly observed in patients with GM-IVH, was also significantly reduced after treatment with VP3.15 (Fig. 3f and g).

### VP3.15 Restores Neuronal Compromise in Animals with GM-IVH

**Fig. 4:**
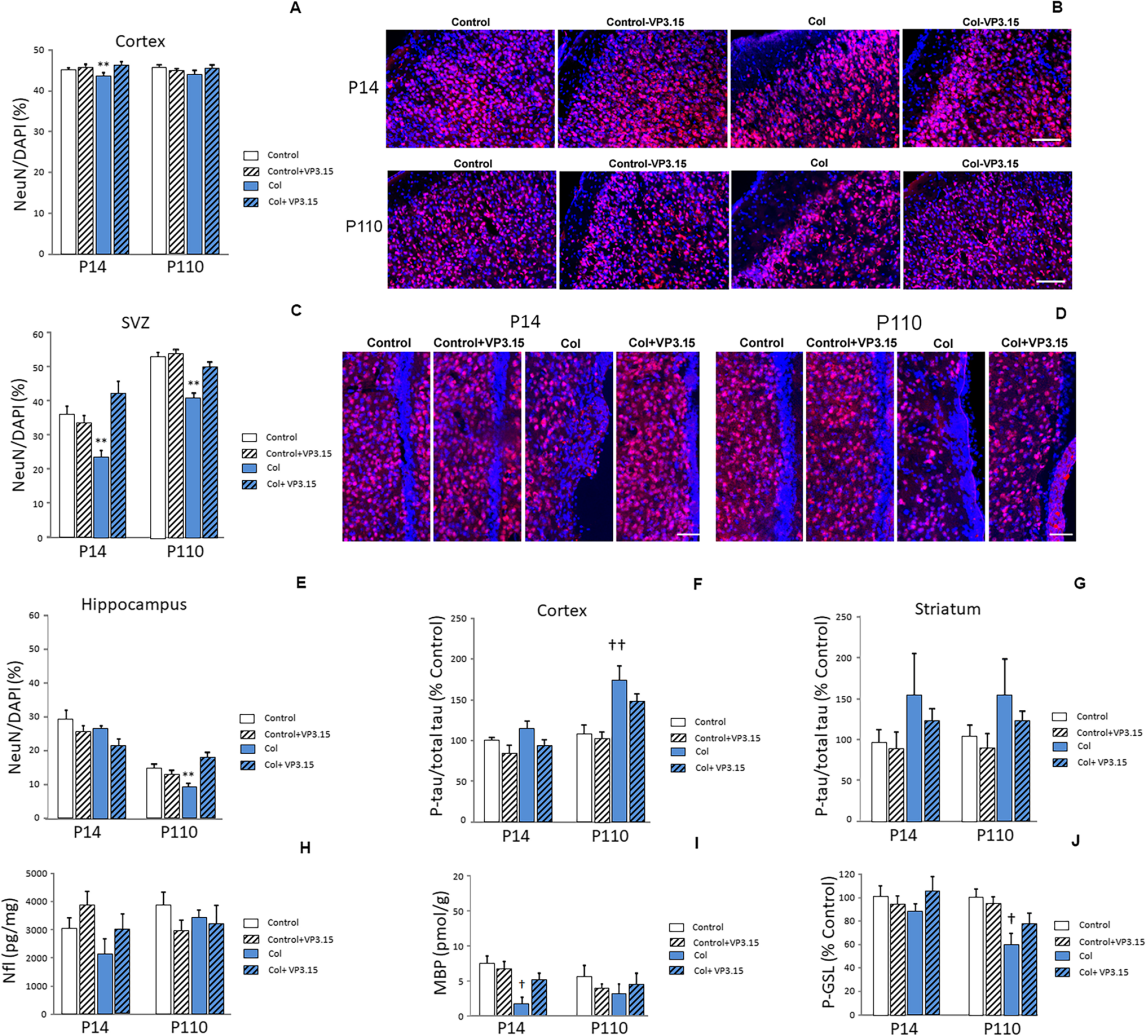
VP3.15 restores neuronal compromise in animals with GM-IVH. **a** The NeuN/DAPI ratio was compromised in the cortex from mice with GM-IVH, and VP3.15 treatment significantly ameliorated this situation at P14 [F_(3,2348)_ = 4.78, **p = 0.003 vs. rest of the groups], although no differences were observed at P110 [F_(3,2363)_ = 0.979, *p* = 0.404]. Data are representative of 5–6 mice (P14: Control *n* = 5, Control + VP3.15 *n* = 6, Col *n* = 5, Col + VP3.15 *n* = 6. P110: Control *n* = 6, Control + VP3.15 *n* = 5, Col *n* = 5, Col + VP3.15 *n* = 6). **b** Illustrative example of NeuN (red)/DAPI (blue) staining in the cortex. Scale bar = 100 µm. **c** The NeuN/DAPI ratio was compromised in the SVZ from mice with GM-IVH, and VP3.15 treatment significantly ameliorated this situation at P14 [F_(3,240)_ = 5.61, **p = 0.001 vs. rest of the groups]. A similar profile was observed at P110 [F_(3,434)_ = 22.69, **p = 0.001 vs. rest of the group]. Data are representative of 3–7 mice (P14: Control *n* = 3, Control + VP3.15 *n* = 5, Col *n* = 4, Col + VP3.15 *n* = 5. P110: Control *n* = 7, Control + VP3.15 *n* = 5, Col *n* = 4, Col + VP3.15 *n* = 4). **d** Illustrative example of NeuN (red)/DAPI (blue) staining in the SVZ. Scale bar = 50 µm. **e** No differences were observed when we analyzed the NeuN/DAPI ratio in the hippocampus at P14 [F_(3,223)_ = 1.91, *p* = 0.128]. A reduction in the NeuN/DAPI ratio was observed at P110, and VP3.15 restored this situation [F_(3,536)_ = 5.34, **p < 0.001 rest of the groups]. Data are representative of 4–7 mice (P14: Control *n* = 4, Control + VP3.15 *n* = 5, Col *n* = 4, Col + VP3.15 *n* = 4. P110: Control *n* = 7, Control + VP3.15 *n* = 6, Col *n* = 7, Col + VP3.15 *n* = 7). **f** The P-tau/total tau ratio was slightly increased in the cortex from P14 with GM-IVH mice, although the differences were not statistically significant [F_(3,18)_ = 2.12, *p* = 0.132]. In the long term, tau hyperphosphorylation was increased in animals with GM-IVH, and VP3.15 ameliorated this situation [F_(3,14)_ = 5.78, ††*p* = 0.009 vs. Control and Control + VP3.15]. Data are representative of 4–6 mice (P14: Control *n* = 4, Control + VP3.15 *n* = 6, Col *n* = 6, Col + VP3.15 *n* = 6. P110: Control *n* = 4, Control + VP3.15 *n* = 4, Col *n* = 5, Col + VP3.15 *n* = 5). **g** The P-tau/total tau ratio was increased in the striatum from P14 with GM-IVH, and animals treated with VP3.15 showed a better profile, although the differences did not reach statistical significance [F_(3,17)_ = 0.997, *p* = 0.418]. A similar profile was observed in the long term [F_(3,17)_ = 1.24, *p* = 0.325]. Data are representative of 4–7 mice (P14: Control *n* = 4, Control + VP3.15 *n* = 6, Col *n* = 6, Col + VP3.15 *n* = 6. P110: Control *n* = 5, Control + VP3.15 *n* = 5, Col *n* = 7, Col + VP3.15 *n* = 4). **h** Nfl levels were slightly reduced in the cortex from P14 mice with GM-IVH, and VP3.15 ameliorated this situation, although the differences did not reach statistical significance [F_(3,20)_ = 1.56, *p* = 0.152]. No differences were observed at P110 [F_(3,20)_ = 0.579, *p* = 0.636]. Data are representative of 6 mice. Data are representative of 6–8 mice (P14: Control *n* = 6, Control + VP3.15 *n* = 6, Col *n* = 8, Col + VP3.15 *n* = 7. P110: Control *n* = 6, Control + VP3.15 *n* = 6, Col *n* = 7, Col + VP3.15 *n* = 7). **i** MBP levels were significantly reduced in the cortex at P14 [F_(3,17)_ = 3.23, †*p* = 0.048 vs. Control]. Whereas a similar profile was observed at P110, the differences did not reach statistical significance [F_(3,15)_ = 0.53, *p* = 0.66]). Data are representative of 5–6 mice (P14: Control *n* = 6, Control + VP3.15 *n* = 5, Col *n* = 5, Col + VP3.15 *n* = 6. P110: Control *n* = 6, Control + VP3.15 *n* = 6, Col *n* = 6, Col + VP3.15 *n* = 6). **j** p-GSL levels were not significantly affected at P14 [F_(3,45)_ = 0.967, *p* = 0.416]. At P110, p-GSL was significantly reduced in animals with GM-IVH, and mice treated with VP3.15 showed no differences from control animals [F_(3,39)_ = 4.19, †*p* = 0.011 vs. Control and Control + VP3.15]. Data are representative of 11–19 mice (P14: Control *n* = 13, Control + VP3.15 *n* = 13, Col *n* = 19, Col + VP3.15 *n* = 12. P110: Control *n* = 11, Control + VP3.15 *n* = 12, Col *n* = 11, Col + VP3.15 *n* = 13)

When we analyzed the neuronal population, we observed that the NeuN/DAPI ratio was compromised in the cortex from mice with GM-IVH at P14. An improvement in NeuN/DAPI ratio was observed after treatment with VP3.15. Differences among groups were no longer observed at P110 (Fig. 4a and b). Neuronal compromise detected in the SVZ at the earliest timepoint was completely reversed by VP3.15, and a similar trend was observed in the long term (Fig. 4c and d). A similar profile was observed in the hippocampus, and treatment with VP3.15 significantly reduced neuron loss at P110 (Fig. 4e).

### Tau Hyperphosphorylation is Reduced in Animals with GM-IVH after VP3.15 Treatment

We detected an increase in pS199 when the cortex from animals with GM-IVH was analyzed at P14 that reached statistical significance at P110 (Fig. 4f). P-tau/total tau ratios were reduced after VP3.15 administration. While differences were not statistically significant when compared with untreated mice, no differences were observed when animals treated with VP3.15 were compared with control mice (Fig. 4f). We did not detect significant differences when we analyzed the striatum (Fig. 4g).

### Nfl Levels are not Affected in Mice with GM-IVH

We observed a slight reduction in Nfl levels in the cortex from mice with GM-IVH at early timepoints, and VP3.15 improved this situation, although the differences did not reach statistical significance. No differences were observed in the long term when Nfl was analyzed in the cortex (Fig. 4h).

### VP3.15 Restores MBP Levels in Animals with GM-IVH

MBP levels, an indicator of the state of the myelin sheath, were significantly reduced in animals with GM-IVH at P14, and VP3.15 successfully limited this effect. Differences were no longer detected at P110 (Fig. 4i).

### VP3.15 Restores p-GLS Levels in Animals with GM-IVH

As previously described, p-GLS levels were reduced in animals with GM-IVH [25], and differences reached statistical significance at P110, while VP3.15 successfully restored p-GLS levels (Fig. 4j).

### Proliferation and Neurogenesis

**Fig. 5:**
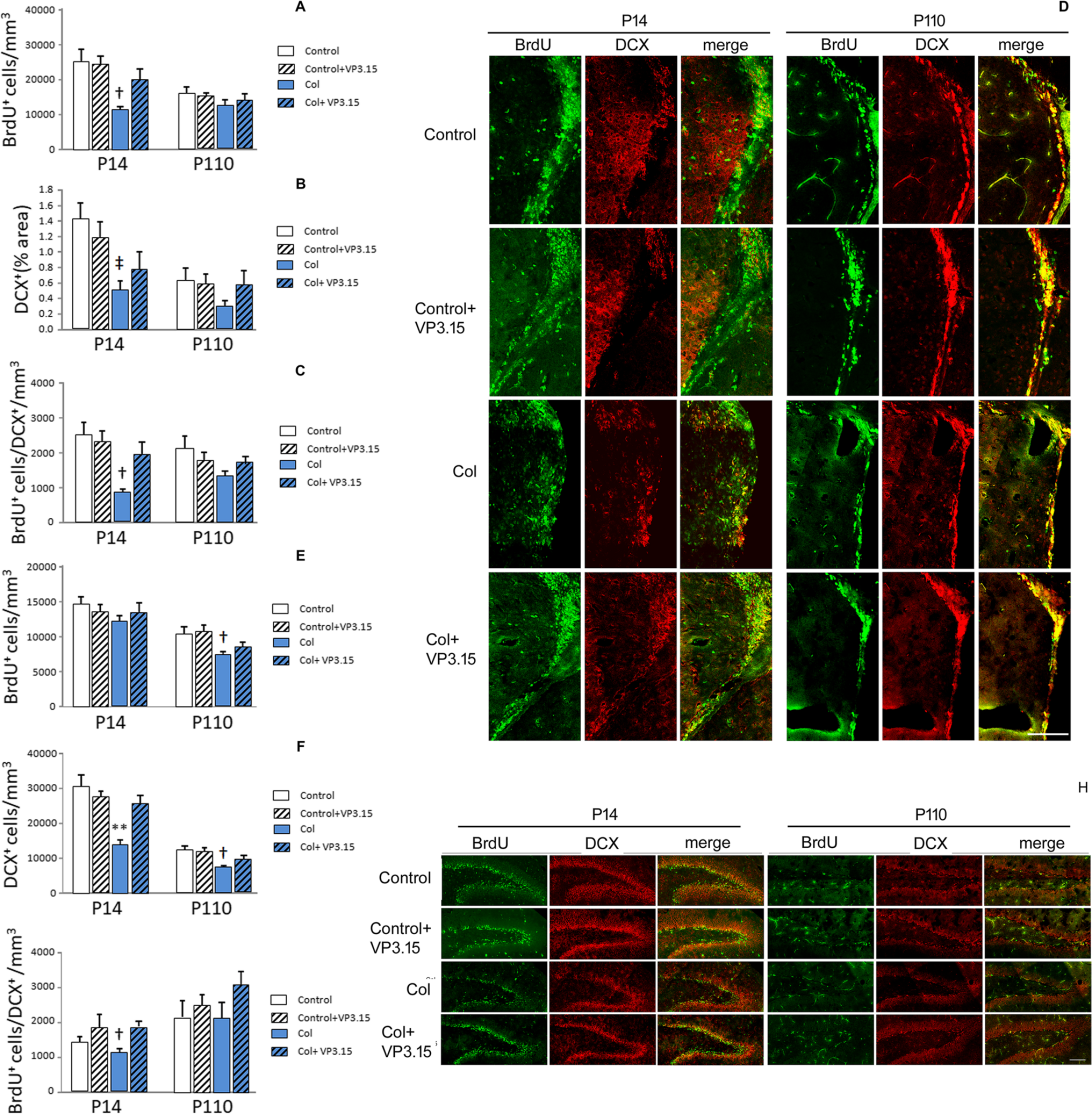
VP3.15 improves proliferation and neurogenesis in mice with GM-IVH. **a** The number of BrdU^+^ cells was significantly reduced in the SVZ from animals with GM-IVH at P14, and VP3.15 treatment increased the number of BrdU^+^ cells [F_(3,53)_ = 3.42, †*p* = 0.026 vs. Control and Control-VP3.15]. Differences were no longer significant at P110 [F_(3,46)_ = 0.756, *p* = 0.524]. **b** The area covered by DCX immunostaining was significantly reduced in the SVZ at P14 [F_(3,53)_ = 3.24, ‡*p* = 0.029 vs. Control]. A similar profile was observed at P110, but the differences did not reach statistical significance [F_(3,51)_ = 0.512, *p* = 0.676]. **c** The BrdU/DCX ratio was significantly compromised in animals with GM-IVH, and VP3.15 improved this limitation at P14 [F_(3,55)_ = 3.12, †*p* = 0.033 vs. Control and Control-VP3.15]. VP3.15 also improved this ratio in the long term (P110), although the differences were not statistically significant [F_(3,51)_ = 1.26, *p* = 0.296]. Data are representative of 4-7mice (P14: Control *n* = 7, Control + VP3.15 *n* = 6, Col *n* = 4, Col + VP3.15 *n* = 4. P110: Control *n* = 6, Control + VP3.15 *n* = 5, Col *n* = 4, Col + VP3.15 *n* = 5). **d** Illustrative example of BrdU and DCX immunostaining in the SVZ from all groups under study. Scale bar = 100 µm. **e** The number of BrdU^+^ cells was slightly reduced in the DG from animals with GM-IVH, although differences were not statistically significant at P14 [F_(3,64)_ = 0.713, *p* = 0.548]. By P110, the number of BrdU^+^ cells was significantly reduced in the DG, and treatment with VP3.15 ameliorated this situation [F_(3,54)_ = 0.30, †*p* = 0.021 vs. Control and Control + VP3.15]. **f** The number of DCX^+^ cells was significantly compromised in animals with GM-IVH in the short term, and VP3.15 completely reversed this situation [F_(3,65)_ = 7.38, **p < 0.01 vs. rest of the groups]. In the long term, VP3.15 treatment restored the number of DCX^+^ cells to control values in the DG from animals with GM-IVH [F_(3,54)_ = 7.05, †*p* = 0.036 vs. Control and Control + VP3.15]. **g** The BrdU^+/^DCX^+^ ratio was also significantly reduced in animals with GM-IVH, while VP3.15 improved this situation at P14 [F_(3,64)_ = 3.38, †*p* = 0.023 vs. Control-VP3.15 and Col-VP3.15]. Differences were no longer significant at P110 [F_(3,54)_ = 1.25, *p* = 0.229]. Data are representative of 4-7mice (P14: Control *n* = 7, Control + VP3.15 *n* = 7, Col *n* = 6, Col + VP3.15 *n* = 6; P110: Control *n* = 6, Control + VP3.15 *n* = 6, Col *n* = 4, Col + VP3.15 *n* = 7). **h** Illustrative example of BrdU and DCX immunostaining in the DG from all groups under study. Scale bar = 100 µm

GM-IVH directly affects the SVZ, and therefore, this neurogenic niche is severely compromised after Col lesions, as previously described [25]. However, VP3.15 favored proliferation, as analyzed by BrdU immunostaining, especially in the short term (Fig. 5a and d). Similarly, the DCX^+^ area was increased in the SVZ after VP3.15 treatment (Fig. 5b and d), and the proliferation/neurogenesis ratio was significantly improved by VP3.15 administration (Fig. 5c and d). These effects were more robust at P14, probably due to the direct damage of the region in the model as well as in patients. To further analyze proliferation and neurogenesis, we also analyzed the DG of the hippocampus. After Col administration, this neurogenic niche was also compromised, and the number of BrdU^+^ cells was reduced in the long term, while VP3.15 ameliorated this limitation (Fig. 5e and h). An overall reduction in the number of DCX^+^ cells was also observed, although VP3.15 successfully increased neurogenesis in the DG of treated mice (Fig. 5f and h). Likewise, the proliferation/neurogenesis ratio was improved by VP3.15 in mice with GM-IVH (Fig. 5g and h). These observations support a beneficial role for VP3.15 in proliferation and neurogenesis.

## Discussion

GM-IVH remains one of the most recurrent and devastating complications of the neonatal period [38], and associated morbidities include intellectual disabilities, cerebral palsy, and neurodevelopmental or psychiatric disorders (for review, see [39]). GM-IVH commonly results in white matter lesions closely linked to the adjacent bleed [38]. Nevertheless, it also induces substantial injury to the cerebral gray matter that further contributes to cognitive deficits and neurobehavioral disorders [39], and the grade of GM-IVH greatly determines the extent of the actual damage.

The sequelae of GM-IVH are also largely dependent on the severity of the lesion and it has been described that may affect up to two-thirds of these children [39]. Since GM-IVH has no successful treatment [40], these patients are in tremendous need of new therapeutic options that might reduce brain damage. VP3.15, a dual inhibitor of GSK-3β and PDE7, has been shown to have neuroprotective activity in other neurodegenerative diseases, such as multiple sclerosis [41] or retinitis pigmentosa [22]. Interestingly, VP3.15 has a direct effect on cerebral grey matter, but it also has remyelinating properties [23, 24], providing a relevant approach for GM-IVH, in which both grey and white matter injuries are regularly observed.

We analyzed the effects of VP3.15 in mice with GM-IVH of the PT by intracerebroventricular unilateral administration of Col to P7 mice [42]. Whereas the model has inherent limitations [3], this animal model mimics many complications observed in GM-IVH, including the presence of hemorrhages, brain atrophy and ventricle enlargement, neuronal loss, proliferation and neurogenesis compromise or increased inflammation that results in learning and memory alterations at later stages of life [14, 17, 25]. Since the severity of the lesions seems to have a direct effect on the seriousness of the developmental disorders in patients [3, 10], a pilot assessment (not shown) revealed that 3 h after Col lesions, hemorrhages developed invading the ventricle and mimicking a grade III or IV GM-IVH. While the study of this time point is of interest, the fact that we wanted to test the effects of VP3.15 led us to complete the studies at P14 as an early time point, immediately after completing the treatment.

We detected an overall increase in the presence of hemorrhages in regions adjacent to the lesion (striatum) and also in further regions, like the hippocampus and the cortex. Previous studies have shown that low-grade GM-IVH results in vascular abnormalities (cerebral blood flow) in the germinal matrix and also in distant regions, including posterior cortical and subcortical gray matter regions [36], suggesting an overspread vascular compromise as well as a regional vulnerability of these developing brain structures. Similarly, other animal models have revealed that the induction of GM-IVH results in an increase of hemeoxigenase-1 expression in the hippocampus and the cortex, and the release of iron from the heme group may play a role in ventricular dilation [37], in line with our observations. VP3.15 effectively reduces the presence of hemorrhages in the cortex, hippocampus and striatum. Whereas to our knowledge, this effect has not been specifically analyzed after VP3.15 treatments, other studies have proposed the beneficial effect of GSK-3β inhibition on blood‒brain barrier integrity [43]. Likewise, other cAMP phosphodiesterase inhibitors, such as rolipram, have shown their capacity to reduce intracerebral hemorrhage in animals [44]. On the other hand, VP3.15 has been shown to reduce mRNA levels of GFAP, COX-2 mRNA and VCAM, an adhesion molecule implicated in the extravasation of leukocytes across the blood‒brain barrier, as well as in CNS inflammatory insults [21]. VP3.15 limits the presence of iron deposits, as a consequence of the induced bleeding. Our postmortem studies preclude the assessment of the direct effects of GM-IVH, but reducing toxic blood products and debris might contribute to improve the outcomes of the lesions, as suggested with other approaches to treat GM-IVH [37, 45].

We detected an increase in the presence of microglia, however, since we only used Iba1 immunostaining, further characterization could provide more dialed information regarding the microglial states and functions after the lesions [46]. We also observed that VP3.15 reduces microglia burden in the cortex, hippocampus and SVZ in mice with GM-IVH. Similarly, previous studies have also reported that VP3.15 positively modulates central inflammation in a murine model of primary progressive multiple sclerosis, showing reduced Iba1 staining in the spinal cord [41]. In line with these observations, other studies have reported that GSK-3β knockdown significantly reduces IL-6 production in astrocytes challenged with lipopolysaccharide [47]. Additionally, PDE7 inhibitors have been considered a feasible treatment for inflammatory and autoimmune diseases [48].

Brain atrophy is largely observed after GM-IVH, and it has been suggested that it might be due to loss of infarcted tissue but also to impaired development of the remaining tissue over time [49]. An overall reduction in brain size was detected, an alteration that was still observed even in young adults, supporting the long-lasting complications associated with GM-IVH of the PT. Animals had smaller cortical sizes, as well as enlarged ventricles, as commonly observed in the clinic [50]. We have previously reported similar damages, and other studies in animals with GM-IVH have also shown overspread harm that includes the cortex [37, 51, 52]. Likewise, cortical alterations are detected in patients [36, 51]. Early reductions in Nfl were not severe enough to result in significant differences. Nevertheless, the neuronal population was compromised in animals with GM-IVH, as previously observed in the model [14, 17, 25], being especially severe in the SVZ, due to its location in the proximity of the lesion. VP3.15 significantly ameliorated this situation, supporting its neuroprotective role. VP3.15 successfully preserves axonal integrity in a model of primary progressive multiple sclerosis, and it has been suggested that the simultaneous inhibition of PDE7 and GSK-3β might account for its synergistic effect [41]. In line with these observations, neuroprotection mediated by inhibition of GSK-3β has been largely reported after different insults and neurodegenerative disorders [53–55], and similar results have been observed after PDE7 inhibition [56]. We detected an increase in tau phosphorylation in the cortex of mice with GM-IVH. As a marker of neuronal damage, increased tau phosphorylation has been detected in different neuropathological insults, and early tau alterations might be enough to affect cognitive function [57]. We also observed that tau hyperphosphorylation was reduced by VP3.15. While the effects were promising, we only analyzed tau phosphorylation at S199 and therefore we cannot exclude that the phosphorylation of other tau residues may play a relevant role. In this sense, previous studies in this model have revealed an increase of tau phosphorylation at S202 and and T205 [25]. Moreover, it is feasible that specific phosphorylated residues might depend on the timepoint analyzed after the lesions or the severity of GM-IVH. Since GSK3 contributes to the hyperphosphorylation of tau protein, GSK-3β inhibitors have been largely used to limit tau phosphorylation in other neurodegenerative disorders [58, 59], and PDE7 inhibitors also reduce tau hyperphosphorylation by cross-talk with GSK-3, increasing its inactive form through PKA phosphorylation [60, 61].

We also detected that proliferation and neurogenesis were compromised in the neurogenic niches (SVZ and DG) from animals with GM-IVH, as observed in patients [55]. The fact that BrdU staining shows an overall reduction, supports that progenitor cells are affected, as it could be expected. Also the BrdU-DCX ratio is reduced in mice with GM-IVH, suggesting neurogenesis is also compromised. DCX is a microtubule-associated protein expressed by migrating neuroblasts and a reliable marker of neurogenesis, however it is also expressed in mature astrocytes [52] and further staining procedures would be required to assess different cell populations that could probably be affected by the lesions. Also, it is feasible that further assessement of the complete periventricular region might provide relevant information regarding the proliferation and neurogenesis processes after GM-IVH. Treatment with VP3.15 succesfully ameliorated this limitation. To our knowledge, no previous study has addressed the effects of VP3.15 on proliferation or neurogenesis in vivo, although other studies have reported the beneficial effects of GSK-3β inhibition after GM-IVH. It has been suggested that this effect might be mediated by stimulation of Wnt signaling to increase β-catenin levels and enhance neurogenesis [55]. Interestingly, PDE7 inhibition also induces proliferation and promotes stem cell differentiation toward a neuronal phenotype [62], an effect that has also been observed in animal models of other neurodegenerative diseases [63].

GM-IVH severely damages white matter, and the effect on oligodendroglial progenitor cells also results in reduced myelination (for review, see [64]). We observed a reduction in MBP when analyzed at early timepoints, and although a similar profile was observed in the long term, differences were no longer detected. Given that white matter damage directly affects the periventricular region, it is feasible that specific analysis of this area would result in more severe depletion of MBP levels. Nevertheless, our observations are in line with other studies showing that staining for MBP is reduced after GM-IVH [65] and that oligodendrocyte lineage proliferation and maturation are interrupted after the lesion, preventing myelination [66]. To our knowledge, the myelination activity of dual GSK-3β/PDE7 inhibitors has not been assessed in GM-IVH of the PT. However, previous studies have shown that VP3.15 reduces demyelinating lesions in experimental autoimmune encephalomyelitis [21]. It also promotes remyelination and oligodendrocyte progenitor cell differentiation in animal models as well as in patients with demyelinating conditions [23, 24, 41]. On the other hand, PDE7 inhibition promotes oligodendrocyte precursor differentiation and survival during brain development and in adulthood [67]. Interestingly, p-GLS was reduced in mice with GM-IVH. Similar results have been reported in this animal model [17, 25] as well as in other complications of the PT [27, 68], supporting its predictive value. The fact that VP3.15 restores p-GLS shows that brain complications of GM-IVH are also observed in the periphery and supports the prognostic value of p-GLS.

Our model of GM-IVH shows learning and memory alterations [14, 25, 34], as observed in patients [69], and VP3.15 limits cognitive impairment. VP3.15 has been previously described to reduce motor deficits in other animal models [41], and specific GSK-3β [70, 71] or PDE7 [63, 72] inhibition has been largely shown to improve cognitive impairment in other disorders. We cannot attribute the observed compromise in learning and memory to a single pathological feature associated with GM-IVH. In the same way, it is feasible that the beneficial effects of VP3.15 in cognition comprise different aspects, including the amelioration of grey and white matter damages, improving neurogenesis alterations and brain atrophy or reducing the inflammatory response.

We cannot obviate that the animal model under study mostly reproduces the most severe grades of GM-IVH and therefore outcomes may differ after mild lesions. Also, while VP3.15 is commonly dissolved in DMSO with beneficial neuroregenerative results [21, 24], this approach is still a limitation for its potential clinical use. Despite this, VP3.15 dual GSK-3β and PDE7 inhibition has shown pleiotropic beneficial effects after GM-IVH, including reduced bleeding, inflammation or grey and white matter compromises. VP3.15 treatment also results in improved cognition in the long term and restored p-GLS levels. Altogether, our data support the promising effects of VP3.15 in reducing complications associated with GM-IVH of the PT.

## Data Availability

Data are available upon reasonable request.

## Abbreviations

BrdU: 5-Bromo-2-deoxyuridin
CNS: Central nervous system
Col: Collagenase
DG: Dentate gyrus
DCX: Doublecortin
GM-IVH: Germinal matrix-intraventricular hemorrhage
GSK-3β: Glycogen synthase kinase-3β
MWM: Morris water maze
MBP: Myelin basic protein
Nfl: Neurofilament light
NOD: New object discrimination test
PDE7: Phosphodiesterase 7
p-GLS: Plasma gelsolin
PT: Preterm infants
SVZ: Subventricular zone
